# Supplementary material for: Assessment of antimalarial activity of crude extract of Chan-Ta-Lee-La and Pra-Sa-Chan-Dang formulations and their plant ingredients for new drug candidates of malaria treatment: In vitro and in vivo experiments
Source: PLoS One. 2024 Jan 11;19(1):e0296756. doi: 10.1371/journal.pone.0296756 (PMC10783769; doi:10.1371/journal.pone.0296756)
Supplement: Attachment — Submitted filename: POINT-BY-POINT.pdf [file pone.0296756.s001.pdf]

POINT-BY-POINT RESPONSES TO THE REVIEWERS' COMMENTS

Editor

Histopathological photographs should be made in a higher magnification, as suggested by Reviewer #2. Several clarifications in Material and Methods, required by both reviewers, are needed as well. Conclusion seems to fail to be sustained by results provided in Table 9. Please provide species authorities for the species listed in L100-102 as well as for *Artemisia vulgaris* in Table 1. In L323, it is Rane's test instead of Rane test.

Response: A manuscript has been revised as suggested.

Reviewer #1:

General comment: This study assessed the antimalarial activity of crude extract of Chan-Ta-Lee-La and Pra-Sa-Chan-Dang formulations and their plant ingredients for new drug candidates of malaria treatment both in vitro and in vivo. The paper is well written and the results well presented. The need for scientific appraisal of antimalarial formulations cannot be overemphasized, hence the study is very relevant.

Response: Thank you.

Specific comments to be addressed:

The author must however revise line 274 "eliminating waste from the cages daily" is not the standard practice. Authors must also cite the international guideline(s) being referred to in line 276.

Response: The sentence has been revised. Please see page 13, line 275.

It is also worthy to note that though the mean parasitaemia level was lower than 90% (which is an indication that both formulations were active as antimalarials) in the in vivo study, with the % suppression of both CTLL and PSCD being less than 50 (Table 9) the formulations cannot be said to have showed a good antimalarial (curative) activity, hence conclusion is not well justified. Authors must revise the conclusion.

Response: The conclusion has been revised. Please see page 43, line 857-859 and page 3, lines 49-51.

Reviewer #2:

• The authors mentioned that "Each plant and formulation 165 were extracted using ethanol and distilled water.". What was the percentage of the used ethanol?

Response: The percentage of ethanol at 95% has been added to the manuscript. Please see page 8, line 165.

• Please mention the exact temperature where room temperature varies from country to country "at room temperature for 72 h…..".

Response: Room temperature at 25 °C has been added to the manuscript. Please see page 9, line 169.

• Please mention the source of *Plasmodium falciparum.*

Response: The sentence about the source of *Plasmodium falciparum* has been added to the manuscript. Please see page 9, lines 182-184.

• For parasitemia determination "The percentage of parasitemia was determined daily using a light microscope": I suggest adding a reference (cite) and calculate the percentage of suppression from The antiplasmodial and spleen protective role of crude Indigofera oblongifolia leaf extract traditionally used in the treatment of malaria in Saudi Arabia. Drug Des Devel Ther. 2015 Nov 25;9:6235-46. doi: 10.2147/DDDT.S94673.

Response: A suggested reference (cite) has been added. Please see page 9, line 190.

• If the used mice were adult (6-8 weeks) add adult before mice.

Response: The word "adult" has been added to the manuscript. Please see page 13, line 268.

• Mention how you have calculated 1×10^7 P. berghei-parasitized RBCs to be the used dose. Mention the used chamber.

Response: For the calculation of 1×10^7 *P. berghei*-parasitized RBCs to be the used dose, we calculated from the level of parasitemia in donor mice. When the level of parasitemia in donor mice reached 20–30%, the donor mouse was subsequently sacrificed and blood collected (via cardiac puncture) into a heparinized tube. Based on the parasitemia level of the donor mouse and

the red blood cell (RBC) count of a normal mouse, the collected blood was diluted with normal saline (0.9%), so that 1 ml of blood contains 5 x 10^7 infected RBCs. Each mouse was injected with 0.2 ml of blood containing 1 x 10^7 *P. berghei*-infected RBCs via the intraperitoneal route.

Ref: Belay, W. Y., Endale Gurmu, A., & Wubneh, Z. B. Antimalarial activity of stem bark of *Periploca linearifolia* during early and established *Plasmodium* infection in mice. J Evid Based Complementary Altern Med.*2018*; 4169397. https://doi.org/10.1155/2018/4169397

• Did you use the whole blood or you separated the parasitized erythrocytes to be only injected into experimental mice?

Response: For the infection of mice, we used whole blood. Briefly, blood from donor mice was collected and then diluted with 0.9% physiological isotonic saline before injected into mice in the experiment.

• In table 10: "Table 10. Percent suppression of the ethanolic extract of the CTLL and PSCD formulations in prophylactic test" mention the day of blood collection. Why this day.

Response:

- The word "Day 6" has been added to the table name. Please see page 30, line 563.
- We monitored parasitemia on day 6 (day 6 after mice received the extract or day 3 post-infection) because the parasitemia level increased steadily beginning on day 3 post-infection. The reason could be that only approximately one-third of infected red blood cells are able to circulate freely in the blood circulation within 48 hours, whereas the remaining parasites stick to the venules and capillaries. Therefore, blood collection at day 3 post-infection would provide a precise assessment of the parasitemia.

Ref: Nwonuma CO, Balogun EA, Gyebi GA. Evaluation of antimalarial activity of ethanolic extract of *Annona muricata* L.: An in vivo and an in-silico approach. Journal of Evidence-Based Integrative Medicine. 2023;28.

• In the histopathology: I cannot see any histopathological change with this magnification. I found no figure caption describing the groups and the histopathological change. I cannot see the scale bar. I also suggest in addition to add a higher magnification to add a histology score.

Response: Thank you for your suggestion. A new figure of histopathological change with a higher magnification has been added.

• I suggest comparing your results with authors used any of the used extracts or mention the activity of other extracts and write on the important of using medithinal plants against malaria. You can use “Medicinal plants as a fight against murine blood-stage malaria. Saudi J Biol Sci. 2021 Mar;28(3):1723-1738. doi: 10.1016/j.sjbs.2020.12.014.

Response: Thank you for your valuable comment. I have already compared results with authors who used any of the used extracts or mentioned the activity of other extracts (please see pages 37–38, lines 720–723) and wrote about the importance of using medicinal plants against malaria (please see pages 34, lines 63–63).
