# Supplementary material for: Assessment of antimalarial activity of crude extract of Chan-Ta-Lee-La and Pra-Sa-Chan-Dang formulations and their plant ingredients for new drug candidates of malaria treatment: In vitro and in vivo experiments
Source: PLoS One. 2024 Jan 11;19(1):e0296756. doi: 10.1371/journal.pone.0296756 (PMC10783769; doi:10.1371/journal.pone.0296756)
Supplement: Attachment — Submitted filename: POINT-BY-POINT.pdf [file pone.0296756.s002.pdf]

POINT-BY-POINT RESPONSES TO THE EDITOR'S REQUEST AND JOURNAL REQUIREMENTS

The Editor's request:

"Please provide species authorities for the species listed in L100-102 as well as for *Artemisia vulgaris* in Table 1.**" You may use Kew Garden's Plants of the World Online (**https://powo.science.kew.org/**).**

Response: We have checked and revised as suggested.

Journal requirements:

Please review your reference list to ensure that it is complete and correct. If you have cited papers that have been retracted, please include the rationale for doing so in the manuscript text, or remove these references and replace them with relevant current references. Any changes to the reference list should be mentioned in the rebuttal letter that accompanies your revised manuscript. If you need to cite a retracted article, indicate the article's retracted status in the References list and also include a citation and full reference for the retraction notice.

Response: We have checked and revised as suggested.
